# Usability Testing of a Bystander Bullying Intervention for Rural Middle Schools: Mixed Methods Study

**DOI:** 10.2196/67962

**Published:** 2025-02-21

**Authors:** Aida Midgett, Diana M Doumas, Claudia Peralta, Matt Peck, Blaine Reilly, Mary K Buller

**Affiliations:** 1 Boise State University Boise, ID United States; 2 Institute for the Study of Behavioral Health and Addiction Boise, ID United States; 3 University of Arkansas Fayetteville, AR United States; 4 Klein and Buendel Golden, CO United States

**Keywords:** technology-based bullying intervention, STAC-T, usability testing, middle school, rural

## Abstract

**Background:**

Targets of bullying are at high risk of negative socioemotional outcomes. Bullying programming in rural schools is important as bullying is more prevalent in those schools compared to urban schools. Comprehensive, school-wide bullying programs require resources that create significant barriers to implementation for rural schools. Because technology-based programs can reduce implementation barriers, the development of a technology-based program increases access to bullying prevention in rural settings.

**Objective:**

We aimed to conduct usability testing of a bystander bullying intervention (STAC-T). We assessed usability and acceptability of the STAC-T application and differences in usability between school personnel and students. We were also interested in qualitative feedback about usability, program features, and feasibility.

**Methods:**

A sample of 21 participants (n=10, 48% school personnel; n=11, 52% students) recruited from 2 rural middle schools in 2 states completed usability testing and a qualitative interview. We used descriptive statistics and 2-tailed independent-sample *t* tests to assess usability and program satisfaction. We used consensual qualitative research as a framework to extract themes about usefulness, relevance, needs, barriers, and feedback for intervention development.

**Results:**

Usability testing indicated that the application was easy to use, acceptable, and feasible. School personnel (mean score 96.0, SD 3.9) and students (mean score 88.6, SD 9.5) rated the application well above the standard cutoff score for above-average usability (68.0). School personnel (mean score 6.10, SD 0.32) and students (mean score 6.09, SD 0.30) gave the application high user-friendliness ratings (0-7 scale; 7 indicates highest user-friendliness). All 10 school personnel stated they would recommend the program to others, and 90% (9/10) rated the program with 4 or 5 stars. Among students, 91% (10/11) stated they would recommend the program to others, and 100% (11/11) rated the program with 4 or 5 stars. There were no statistically significant differences in ratings between school personnel and students. Qualitative data revealed school personnel and students found the application useful, relevant, and appropriate while providing feedback about the importance of text narration and the need for teacher and parent training to accompany the student program. The data showed that school personnel and students found a tracker to report different types of bullying witnessed and strategies used to intervene by students a useful addition to STAC-T. School personnel reported perceiving the program to be practical and very likely to be adopted by schools, with time, cost, and accessibility being potential barriers. Overall, findings suggest that the STAC-T application has the potential to increase access to bullying prevention for students in rural communities.

**Conclusions:**

The results demonstrate high usability and acceptability of STAC-T and provide support for implementing a full-scale randomized controlled trial to test the efficacy of the application.

## Introduction

### Background

National statistics indicate that bullying is a national public health issue in the United States, with 19.2% of students aged 12 to 18 years reporting being bullied at school in the past year [1]. Bullying peaks in middle school, with 26.5% of sixth-grade students reporting being a target of school bullying, followed by 26.3% of seventh graders and 25.1% of eighth graders. Among students who report being bullied, 21.6% report being bullied online. Findings from a meta-analysis examining consequences of bullying have indicated that students who are targets of bullying report a wide range of negative mental health outcomes, including symptoms of anxiety, posttraumatic stress, depressive symptoms, nonsuicidal self-injury, suicidal ideation, and suicide attempts [2]. Similarly, being a target of cyberbullying is associated with internalizing symptoms, suicidal ideation [3-5], and alexithymia and psychotic experiences [6]. Thus, it is imperative to develop effective interventions for middle school students that are accessible and easy to implement to reduce bullying and the associated negative consequences.

### Youth in Rural Schools

Students attending school in rural communities are at high risk of experiencing both school bullying and cyberbullying [7-9]. According to US national statistics, the highest rates of bullying among rural youth in the past decade were reported in 2019, with 27.7% or rural students reporting being bullied compared to 22.4% of students in urban areas [10]. Although rates of bullying peaked in 2019 for both rural and urban students, the most recent US national statistics indicate that the prevalence of school bullying victimization continues to be higher among students in rural areas (23.8%) than among students in urban areas (19%) [1]. Furthermore, among targets of bullying, students attending rural schools are also more likely to report being bullied online (23%) compared to students attending urban schools (19.5%). Rural students also report a higher rate of being bullied with repetition (18.8%) compared to urban students (14.4%). Among middle school students attending schools in rural communities, bullying victimization is associated with poor school relationships, negative school experiences [11], and depression and anxiety [11,12]. These data suggest the importance of developing school-based bullying prevention programs specifically for students in rural communities.

### School-Based Bullying Interventions

Comprehensive, school-wide interventions are effective in reducing bullying and the associated negative mental health outcomes [13]. Furthermore, bystander training (eg, teaching students who witness bullying to intervene in bullying situations) is an important intervention component [13]. Although up to 80% of students report witnessing bullying [14], only 20% intervene [15]. Because students report that they do not know how to intervene when they witness bullying [16], bystander training is a promising approach to bullying prevention. However, few comprehensive school-based programs incorporate bystander training. In addition, comprehensive, school-wide bullying prevention programs are expensive, complex, and time intensive and require extensive training [17]. Because these interventions require substantial resources, many schools face implementation barriers. Schools in rural communities may also face economic disparities, creating further implementation challenges [18], including a lower tax base, increased training costs due to bringing in expert trainers, frequent staff turnover, school closures, staff overload, and lack of program advocates in bullying prevention [19]. Challenges related to logistical problems, training requirements, and limited funding can negatively impact program adoption and sustainability [19].

### Shifting to a Technology-Based Bullying Intervention

Technology-based interventions have the potential to improve access to programming and decrease implementation barriers experienced in rural communities [19]. Although some rural areas have higher rates of poor internet connectivity, eligible schools in rural communities can receive discounts for internet and broadband services [20]. Federal grants to build broadband infrastructure in rural areas are also available [21]. In addition, research conducted with key middle school personnel (ie, administrators, teachers, and school counselors) in rural communities indicates both a strong interest in technology-based bullying prevention programs and positive implementation conditions (eg, administrative support and technology readiness) [22]. Thus, most students in rural communities have access to the necessary infrastructure to support technology-based programs, and key personnel in rural middle schools indicate that schools are interested and ready for technology-based bullying interventions.

Although there is strong interest and need for online bullying prevention programming, very few US bullying prevention programs include a technology component. Programs that do offer it as an adjunct to in-person delivery [13]; often rely on simple texting, not multimedia interfaces; and they do not train student bystanders to intervene. For example, the Build Respect, Stop Bullying program for middle schools uses an online platform [23] but is part of a large program with staff or family components without bystander training. Other available technology programs include (1) an SMS text messaging program pairing youth with a “text buddy” [24]; (2) apps that encourage students to report cyberbullying, block websites attracting cyberbullying, and notify parents and school personnel of cyberbullying; and (3) online social media campaigns and educational resources (eg, videos, testimonies, and quizzes) [18,25,26]. Although there are programs that incorporate technology into bullying prevention and intervention, none appear to offer a route to a technology-based, interactive bystander training for middle school students.

### The STAC Intervention

STAC [27] is a brief, stand-alone bystander intervention that includes didactic and experiential training followed by 2 booster sessions. The 75-minute didactic training includes education about bullying and cyberbullying, the consequences of bullying, and bystander roles and a description of the four STAC strategies: (1) “Stealing the show”—using humor or distraction to interrupt the bullying situation removing the attention away from the target, (2) “Turning it over”—informing an adult about the bullying and asking for help, (3) “Accompanying others”—befriending or providing support to the targeted student, and (4) “Coaching compassion”—gently confronting the perpetrator to increase empathy for the target. The experiential training comprises a series of role-plays during which students practice using the STAC strategies through bullying scenarios. The STAC training is followed by two 15-minute booster sessions to reinforce learning. The STAC intervention is effective in reducing bullying [28,29] as well as mental health risks for bystanders [30-34]. STAC has also been adapted to be culturally appropriate for middle school students in rural communities [35-37]. Research on the adapted STAC intervention demonstrates both bullying reduction [35,38] and improved mental health [35,39] among students trained in the program.

### The Technology-Based STAC Intervention

The technology-based STAC intervention (STAC-T) is an online application developed to shift intervention delivery from in-person implementation to a technology-based format, thereby increasing accessibility and reducing barriers to intervention implementation. STAC-T is designed to be easily disseminated to large groups of students, who can access the intervention from a computer, tablet, or smartphone. In addition, the 40-minute STAC-T application is designed to be modular, increasing implementation flexibility. The initial training is followed by one 15-minute booster session designed to reinforce skill acquisition through virtual role-plays. The program is interactive, including knowledge checks, personalized feedback, and the selection of avatars to respond to bullying scenarios. Initial development included the design and testing of a STAC-T prototype. The design of STAC-T was developed based on the content of the in-person STAC intervention for rural middle schools as well as feedback from an expert advisory board and key middle school personnel in rural communities. In addition, students attending rural middle schools participated in 3 iterative focus groups, providing feedback on program usefulness, content, and functionality [40]. Once developed, the STAC-T prototype was evaluated through usability testing, which provided feedback from end users on program functioning [41]. The results from usability testing with key personnel and students from 2 rural middle schools indicated that the STAC-T prototype was easy to use, acceptable, and feasible, supporting the full-scale development of the STAC-T application [40].

### This Study

Bullying is a significant public health concern for students attending rural schools [7-9]. Comprehensive bullying intervention programs that incorporate bystander interventions are the standard for practice [13]; however, they place a high demand on schools for implementation [17] and can contribute to disparities in rural schools [19-22]. STAC-T has the potential to reduce barriers and increase access to bullying prevention for middle school students in rural settings [40]. Therefore, the purpose of this study was to evaluate the usability and acceptability of the full-scale STAC-T application to determine readiness for a large, multisite randomized controlled trial to evaluate the efficacy of the STAC-T application for middle school students in rural communities. Usability testing is an important step in the process of intervention development as it predicts the likelihood of program adoption [42]. To achieve this aim, we implemented usability testing with key stakeholders (ie, school personnel and students) at 2 middle schools in rural communities in 2 states (N=21) using a mixed methods design. This study had the following objectives: (1) to assess usability and acceptability of the STAC-T application and (2) to assess differences in usability between school personnel and students.

## Methods

### Participants

Participants were key school personnel (ie, administrators, teachers, and school counselors; 10/21, 48%) and students (11/21, 52%) recruited from 2 middle schools in rural, low-income communities in the Northwestern and Southern regions of the United States. Between 5 and 10 usability testers are needed to identify most usability issues [43]. The schools were selected based on previous and ongoing research partnerships. The 2 schools were Title 1 schools, with 95% and 99% of the student population at the 2 schools eligible for reduced or free lunch. Among school personnel, the ages ranged from 26 to 55 years (mean 43.4, SD 9.8 years), and most (9/10, 90%) were female. School personnel self-reported ethnicity or racial background as White (5/10, 50%), Hispanic or Latino (3/10, 30%), and Black or African American (2/10, 20%). Among students, ages ranged from 11 to 15 years (mean 12.8, SD 1.3 years), with 36% (4/11) in grade 6, a total of 18% (2/11) in grade 7, and 45% (5/11) in grade 8. Students self-reported gender as female (6/11, 55%) and male (5/11, 45%). Students self-reported ethnicity or racial background as White (4/11, 36%), Black or African American (4/11, 36%), and Hispanic or Latino (3/11, 27%).

### Development of the STAC-T Application

The translation from the STAC in-person intervention to STAC-T was guided by persuasive system design, a theoretical guide for translating clinical aims to health-related technology frameworks [44-46]. The STAC-T application was developed using Agile programming, a collaborative and incremental programming methodology [44-46]. The application was functional on all web browsers that support HTML5 and was built on a full-stack web application using HTML and JavaScript as the main interface. React.js was used as the front-end framework. The look and feel of the program were designed using Adobe Illustrator and Photoshop and developed using HTML elements plus SVG, PNG, JPG, WAV, MP4, and GIF images, audio, and video graphics. The system is accessible on desktop computers and iOS and Android tablets and smartphones. All design and programming elements were aligned, and stakeholders’ inputs were incorporated throughout the multistage development. Programmers produced the STAC-T application; alpha and beta tested it in-house for stability and code errors; tested it for usability; and revised it following an iterative, Agile production process.

**Figure 1:**
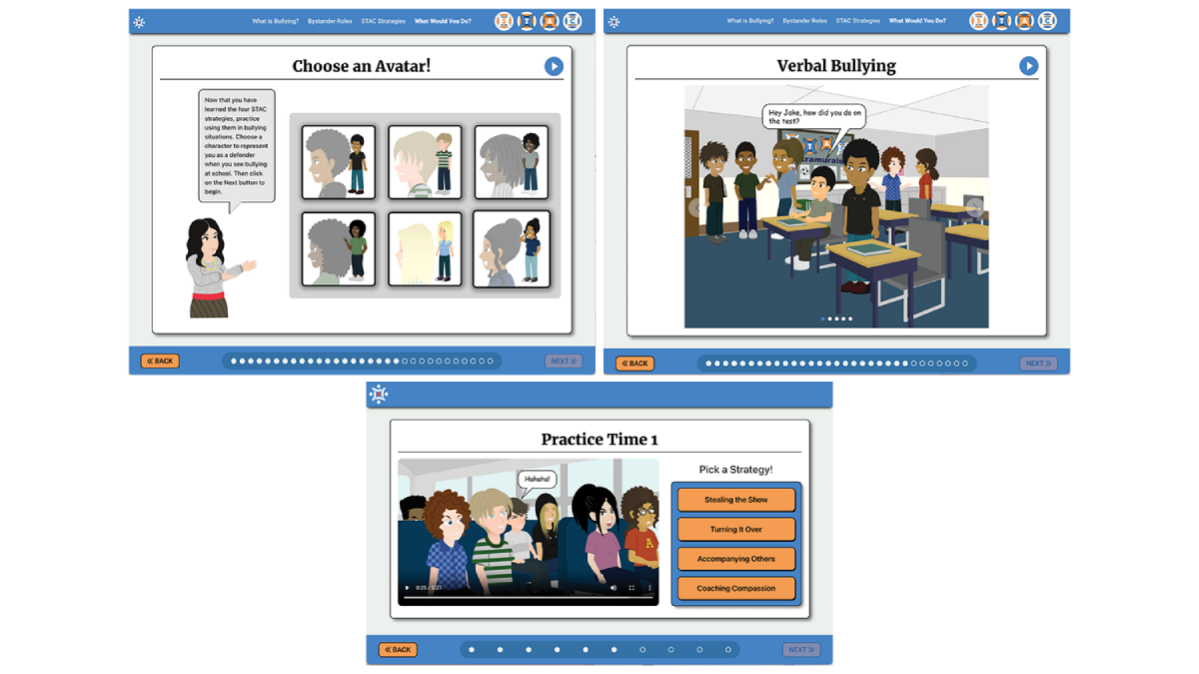
Samples from the technology-based STAC application.

In previous studies, as well as the in the iterative interviews and focus group conducted in this study, participants indicated that increasing program interactivity, adding more color, and including more realistic images such as avatars were important to increase engagement [40] and promote behavior change [47]. Therefore, design elements such as space (bright colors and visual space), components (realistic characters and familiar objects), and mechanics (actions reported by students that occur in rural middle schools) were established for the program features. In addition, STAC strategy practice was designed to require students to select an avatar, view bullying scenarios and select actions to operationalize the STAC strategy, view the avatar enacting the selected action, and receive feedback on its effectiveness. An artist hand illustrated and styled 6 avatars. The avatars had light-, medium-, and dark-colored hair in different styles as well as light, medium, and dark skin tones for students to choose from to best represent themselves and stimulate engagement. To reward learning and bolster adherence, “badges” (visual reward icons; eg, “Show Stealing Badge”) were included as intermittent awards to encourage user engagement (Figure 1).

The STAC-T application content comprises three modules: (1) What is Bullying?—users are presented with background information on bullying, including bullying definitions (ie, physical, verbal, and relationship bullying as well as cyberbullying), bullying facts and statistics, characteristics of students who bully, and negative consequences of bullying; (2) What are Bystanders?—users are taught what a bystander is and how bystanders affect bullying outcomes (this module explains the 4 bystander roles: *assistants*, those who intentionally help the bully; *reinforcers*, those who are not directly involved in hurting another student but encourage the bully by standing around, laughing, or watching quietly; *outsiders*, those who do not take sides while witnessing bullying; and *defenders*, those who do something to stop the bullying situation or help the target in some way); and (3) STAC Strategies—users are introduced to the 4 STAC strategies, which are “Stealing the show,” “Turning it over,” “Accompanying others,” and “Coaching compassion” (this module also includes STAC strategy practice using avatars selected by the user). The booster session includes additional practice with bullying scenarios and STAC strategy use.

Iterative interviews (15/21, 71% of the participants; 7/15, 47% female and 8/15, 53% male; 6/15, 40% White; 6/15, 40% Black or African American; 3/15, 20% Hispanic or Latino) and 2 rounds of iterative focus groups (20/21, 95%; 11/20, 55% female and 9/20, 45% male; 6/20, 30% White; 9/20, 45% Black or African American; 3/20, 15% Hispanic or Latino; 2/20, 10% other) conducted with middle school students attending schools in rural communities in 2 states informed program development before usability testing. Students participating in the interviews provided feedback on design aspects of the program, including color scheme, narration, and cartoons. Students were given a sample slide in 5 color schemes, 3 narrator voice samples, and 3 cartoon-style character depictions. Students were asked to rank a series of questions about each program aspect and then rank their preferences. Feedback and ranked choices were used to select color schemes, the narrator, and the program artist. Iterative focus groups were then conducted to gather feedback from students related to content and stylistic aspects of the program. Overall, the program was well received; students reported that the content was helpful and they liked the look and feel of the teacher who appears throughout the training. Students in the first round of focus groups provided specific feedback to incorporate more cyberbullying scenarios (eg, having bullies use their phones to record their peers without their knowledge), make the appearance of the characters more realistic (eg, changing clothing, adding eyes to all the characters, and changing hairstyles), and improve the function of the program to make navigation more user-friendly. Students also expressed disliking a particular activity, which was removed from the program. Students’ feedback was incorporated into the program before conducting the second round of focus groups. The students in the second round of focus groups provided additional feedback about how to make the appearance of the characters more realistic (eg, adding emotion to the characters), as well as adding background images to make the scenarios look more like what they are used to seeing at school and school-related activities, such as sporting events (eg, adding teachers, lockers, wall hangings, and bulletin boards). They also provided specific feedback about how to make student behaviors more realistic (eg, having the target look sad instead of crying and changing the type of bullying from physical to verbal in front of adults). Input from the focus groups informed the development of the final STAC-T application used in this study*.*

### Procedures

Participant recruitment and usability testing occurred in 2024. The researchers provided the liaisons (eg, school counselor and principal) from each school with an email script describing the purpose and procedures of the study. Inclusion criteria for school personnel consisted of being employed as a principal, teacher, or school counselor at the partnering school; speaking English or Spanish; consenting to participate; and having a desire to make a positive difference in the school climate. For students, inclusion criteria consisted of being enrolled in the partnering school, speaking English or Spanish, having parental consent and student assent, and having a desire to make a positive difference in the school climate. To assess desire to make a positive difference, school liaisons were provided with rubrics developed by the research team to identify key school personnel and students who exhibited the following characteristics assessed by the rubric: caring for students; having a desire to be a positive influence on the school climate; being approachable to students; caring about addressing the problem of bullying; and having leadership qualities in the case of school personnel or leadership, maturity, responsibility, caring toward others, influence, and a desire to be a positive influence on their peers in the case of students. For each item, school personnel and students were assessed on a 3-point scale, which included the ratings of *yes*, *somewhat*, or *no* for each item described previously. School personnel and students who scored *yes* or *somewhat* on all inclusion criteria were eligible to participate. Exclusion criteria for the study for both school personnel and students included having participated in a previous STAC study, speaking a language other than English or Spanish, and not providing consent or assent to participate.

The school liaison used the inclusion and exclusion criteria and the rubric to identify and contact key school personnel and students and then used the script to invite them to participate in the study.

Similarly to our previous usability research [40], we conducted the usability testing and interviews remotely. Research supports remote usability testing as a viable approach to gather high-quality user experience feedback [48,49]. To mitigate technology-related problems, before the testing session, a team member worked with the school liaison to ensure that they could open and operate the program using the school’s computers and firewall. When problems occurred during the testing session, participants switched to a different device or, in one case, rescheduled the testing session.

During the usability testing, participants were asked to review the entire STAC-T application, including the booster session. Participants were asked to talk aloud while completing the tasks, identifying problems and the solutions attempted. The researchers and users were on videoconference and shared their screens. The researchers could see what the participants were doing, and they were able to communicate with each other in real time. The researchers observed the users as they worked through the tasks and asked questions to gather more data. After completing the STAC-T application, participants were asked to complete a brief usability survey followed by a semistructured interview and then a demographic questionnaire. All participants were asked to provide information about their perceptions of (1) program utility, (2) relevance and appropriateness of program content, (3) ways in which they would improve the program, and (4) using a bullying and strategy use tracker after the training. School personnel were also asked about (1) their thoughts on implementation feasibility, (2) the likelihood of school program adoption, (3) their thoughts on companion trainings for teachers and parents, and (4) barriers to program use. All individual interviews lasted 1 hour and were video recorded.

### Measures

#### Demographics

Participants self-reported their age, ethnicity or race, and gender. Students also reported their grade level.

#### Program Usability

Usability was assessed using the System Usability Scale (SUS) [50]. The SUS is a widely used 10-item validated tool that measures the usability and acceptability of technology-based programs. Responses are measured on a 5-point Likert scale ranging from 1 (*strongly disagree*) to 5 (*strongly agree*). To calculate the SUS score, responses are converted as follows: (1) for odd-numbered items, 1 is subtracted from the response; (2) for even-numbered items, 5 is subtracted from the response; (3) the converted responses are added; and (4) the total is multiplied by 2.5. The final SUS score ranges from 0 to 100. An SUS score of ≥68 is considered above average [51].

#### Program User-Friendliness

One item selected from previous usability research [40] was used to assess the user-friendliness of the program. Participants were asked to rate the user-friendliness through the following question—“Overall, I would rate the user-friendliness of this program as:”—using a 7-point scale ranging from 0 (*worst imaginable*) to 7 (*best imaginable*).

#### Program Satisfaction

In total, 2 items selected from previous usability research [40] were used to assess program satisfaction. Participants were asked the following question—“Would you tell your friends/colleagues to use the program?”—with *yes*, *no*, and *don’t know* as response choices. Participants were also asked how many stars they would give the program (1 star being the lowest and 5 stars being the highest).

#### Interview Questions

Following usability testing sessions, participants were asked a series of open-ended questions about the utility and relevance of the application prototype, as well as ways to improve the application, likelihood of program use, and potential implementation barriers. School personnel and students were asked the following: (1) “Please talk about your perception of how useful this program could be to helping to address the problem of bullying at school,” (2) “Please share your thoughts on whether you think the content of this program is relevant and appropriate for students at your school and your community,” and (3) “Can you talk about ways that you would improve the program?” Students were also asked the following: “If your school asked you to continue using the tracker, would it be useful?” School personnel were also asked the following: (1) “What are your thoughts on how practical or workable you think it would be to use this program at your school?” (2) “What do you believe is the likelihood that your school would use this intervention?” (3) “Do you think an online, brief teacher training and parent training module would be a helpful addition to this program?” (4) “What, if anything, would keep you from using this program?” (5) “How would you envision using the tracker, if at all, after completing the training modules and the two boosters?”

### Data Analysis

#### Quantitative Analysis

Quantitative data from the questionnaires were analyzed using SPSS (version 29.0; IBM Corp). Before conducting statistical analyses, we examined the data for missing data points. We found no missing data. The data were also examined for outliers, defined as >3.3 SDs above the mean [52]. We found no outliers. To ensure that continuous data met statistical assumptions for parametric statistical tests, we assessed acceptable normality by using established guidelines for examining skew and kurtosis [53]. All continuous variables were within the acceptable range for skew and kurtosis. Descriptive statistics were used and are presented separately for school personnel and students. We examined differences between school personnel and students using 2-tailed independent-sample *t* tests for continuous variables and chi-square analyses for categorical variables. All statistical assumptions were met for the *t* tests and chi-square analyses. We controlled for type I error using the Bonferroni correction. On the basis of the calculated Bonferroni correction, all analyses were considered significant at *P*<.004.

#### Qualitative Analysis

Qualitative data from open-ended questions were analyzed separately for school personnel and students. In total, 3 team members, 2 of whom conducted the usability tests, transcribed the data verbatim. We used consensual qualitative research as a framework for data analysis. We used thematic analysis [54,55] to identify, analyze, organize, describe, and report themes found within the qualitative data. A faculty member with expertise in qualitative data analysis, along with 2 graduate students, 1 PhD student and 1 masters of art in counseling student, with previous experience in qualitative data analysis, analyzed the data. The faculty member led the data analysis team. The team met 2 times via Zoom (Zoom Video Communications). During their first meeting, the faculty member discussed the analysis protocol with the 2 students, as well as expectations and biases that they needed to be aware of as they analyzed the data. Each team member analyzed the transcripts for the school personnel and students separately to arrive at initial themes for each open-ended question from the interview protocol. Next, the team met one more time via Zoom and conducted additional email communication over a 4-week period to arrive at a consensus on themes and frequency categories supported by participant quotations. During their meetings, the team members shared their themes for each question and discussed agreement or disagreement about themes. The analysts relied on participant quotes to resolve disagreements. Once the team members reached a consensus, an external auditor reviewed the interview transcripts and themes for the school personnel and students. The auditor agreed with the team’s findings. Interview data were deidentified to ensure anonymity, and quotes were identified by participant type (ie, school personnel or students).

### Ethical Considerations

This study was registered with ClinicalTrials.gov (NCT05572398). All research procedures were approved (101-SB21-205) by Boise State University’s institutional review board. Researchers obtained informed consent from school personnel and parental consent and student assent in the case of students. For students, school liaisons met briefly with potential participants identified through the inclusion criteria to explain the project, and interested students were sent home with a letter describing the project as well as an informed consent form for the parent or guardian to sign. English and Spanish translations were provided in schools with a large Hispanic student population. Parents were also emailed the information and consent form. Parents could choose to sign the consent form via pen and paper or electronically. If they did not provide consent electronically, students were asked to return the signed consent form to the school liaison. Parents were provided with the study principal investigator’s contact information and were encouraged to contact her if they had any questions or concerns. Students who returned the signed consent form were then provided with an opportunity to assent immediately before the interview, focus group, or usability testing session. In addition, several steps were taken to protect confidentiality: participants were informed that they were free to refrain from answering any questions, all data were identified only by a personal identifier number, and all research team members completed required training in protection of human research participants. School personnel received a US $50 Amazon gift card as an incentive for participation in the usability testing and individual interview. There were no incentives for student participants.

## Results

### Quantitative Analysis

#### Program Usability

Usability scores on the SUS are presented in Table 1. Overall, scores for both school personnel and students suggested a very high level of usability, functionality, and acceptability. As shown in Table 1, there were no differences in the scores on any of the individual items or the SUS total score between school personnel and students, with both participant groups scoring the STAC-T application at a very high level of usability.

**Table 1:** Means and SDs of the System Usability Scale scores from school personnel and students^a^.

	School personnel (n=10), mean (SD)	Students (n=11), mean (SD)	*t* test (*df*)	*P* value
“I think that I would like to use the program frequently.”	4.60 (0.52)	4.18 (0.60)	–1.70 (19)	.11
“I found the program to be more complex than it needed to be.”	1.20 (0.42)	1.64 (0.92)	1.37 (19)	.19
“I thought the program was easy to use.”	4.90 (0.32)	4.91 (0.30)	0.07 (19)	.95
“I think that I would need the support of a technical person to be able to use this program.”	1.10 (0.32)	1.36 (0.67)	1.13 (19)	.27
“I found the various functions in the program were well put together with each other.”	4.40 (1.27)	4.55 (0.69)	0.33 (19)	.74
“I thought there was too much inconsistency in this program.”	1.00 (0.00)	1.55 (1.04)	1.66 (19)	.11
“I imagine that most people would learn to use this program very quickly.”	4.90 (0.32)	4.55 (0.82)	–1.28 (19)	.22
“I found the program very awkward to use.”	1.00 (0.00)	1.18 (0.40)	1.42 (19)	.17
“I felt very sure that I could use the program correctly.”	4.90 (0.32)	4.55 (0.69)	–1.49 (19)	.15
“I needed to learn a lot of things before I could get going with this program.”	1.00 (0.00)	1.55 (0.93)	1.84 (19)	.08
System Usability Scale total score	96.00 (3.94)	88.64 (9.51)	–2.27 (19)	.04

^a^Responses were scored on a 5-point Likert scale ranging from 1 (*strongly disagree*) to 5 (*strongly agree*).

#### Program User Friendliness

School personnel and students rated the program highly on user-friendliness. Among school personnel, scores on user-friendliness ranged from 6.00 to 7.00 (mean 6.10, SD 0.32). Among students, scores on user-friendliness ranged from 6.00 to 7.00 (mean 6.09, SD 0.30). There were no differences in scores between school personnel and students on user-friendliness (t_19_=–0.07; *P*=.95).

#### Program Satisfaction

Program satisfaction ratings are presented in Table 2. Overall ratings suggested that school personnel and students were satisfied with the program. There were no differences in scores between school personnel and students on program recommendation (*χ*^2^_1_=1.0; *P*=.33) or star ratings (*χ^2^*_1_=2.8; *P*=.25).

**Table 2:** Program satisfaction results for school personnel and students.

			School personnel (n=10), n (%)		Students (n=11), n (%)
**Would recommend the program**					
	Yes	10 (100)		10 (91)	
	No	0 (0)		0 (0)	
	Unsure	0 (0)		1 (9)	
**Star rating**					
	1 star	0 (0)		0 (0)	
	2 stars	0 (0)		0 (0)	
	3 stars	1 (10)		0 (0)	
	4 stars	5 (50)		3 (27)	
	5 stars	4 (40)		8 (73)	

### Qualitative Analysis

#### Overview

Qualitative feedback for the STAC-T application supported the quantitative findings and was very positive overall, with both school personnel and students sharing the perception that the STAC-T application is useful, relevant, and appropriate, as well as providing feedback on ways to improve the program. In addition, school personnel shared positive thoughts about program practicality and adoption, including interest in a teacher and parent training, and discussed barriers to program adoption. Both school personnel and students talked about the benefits of tracking students’ reports of different types of bullying and strategies they used to intervene both as part of the program and as a stand-alone feature to be used after the training. The results are presented in the following sections organized by the following themes: (1) usefulness, (2) relevance and appropriateness, (3) program improvement, (4) program tracker, (5) practicality and adoption, (6) teacher and parent training, and (7) barriers.

#### Usefulness

All school personnel (10/10, 100%) and students (11/11, 100%) indicated that the program was useful and increased students’ knowledge to intervene in bullying situations. For example, a school personnel member shared the following:

...the program that you guys are creating, will definitely inform the students what they need to look for, how they can become an active positive person.

A student also stated the following:

I think it’ll be helpful by telling other kids that it’s not right to bully other kids because you don’t know how they feel, and you don’t know what they go through.

In terms of increasing knowledge, a school personnel member indicated the following:

I like how it has all the different ways for the students to see how you can step in you know...that there’s things they can do.

Another one added the following:

...it gives students the tools that they might feel like they lack in general when bullying happens.

A student stated the following:

It can teach ways on how to and when bullying is happening, how do we handle it, and make it stop quicker.

Another student added the following:

...if people are making fun of a person about how they look or the way they eat, and they post a video on social media, I can like easily screenshot, show it to the principal, my teachers, to get these people to stop and like get them to stop the bullying.

#### Relevance and Appropriateness

All school personnel (10/10, 100%) and students (11/11, 100%) reported that the program content was relevant and relatable and taught students empathy and prosocial attitudes. For example, one school personnel member shared the following:

I think the content was really relevant. It’s things that you actually see at school or that you hear about or that we, that get actually reported.

Another school personnel member added the following:

Oh yeah, absolutely. I think that they [students] can relate to cafeteria situations, getting on the bus and you know posting things especially you know on social media or on Instagram or TikTok or whatever.

A student shared the following:

I think that is really, really relevant and I think that a lot of the situations that were used in this app as examples can be used. They can be real life situations.

In terms of empathy and prosocial attitudes, a school personnel member said the following:

I think this program would help...teach more empathy cause when students have more empathy, they’re less likely to exhibit those bullying behaviors.

A student indicated the following:

It basically says that bullying is not okay and if you do see it here are a few ways on how to stop it.

#### Program Improvement

Both school personnel (9/10, 90%) and students (6/11, 55%) offered feedback on how to improve the program and talked about the importance of having the program be fully narrated. School personnel talked about ways to improve student engagement and user experience. For example, a school personnel member stated the following:

...making it so that all of the pop-ups and scenes are narrated.

A student also said the following:

Once you add that narrative it is going to be good because most middle schoolers they aren’t going to want to read it.

In terms of improving engagement and user experience, a school personnel member said the following:

Just there was one [activity] where you had to click the arrows to move on and I feel like just making it a little bit more simple.

Another school personnel member added the following:

Slowing down the captions on the cartoons because to allow kids to see the picture to get a frame of mind of what’s going on and then read the words.

#### Program Tracker

All the school personnel (10/10, 100%) and students (11/11, 100%) indicated that they would find using a bullying and strategy use tracker useful if their school asked them to continue to use it. When asked about how they would envision using the tracker, school personnel indicated that they would use it for data collection for programmatic feedback and prevention as well as ongoing teaching and student support. For example, when asked about the usefulness of the tracker, a school personnel member stated the following:

Yeah, you know, I think that would be good.

Another one added the following:

You can bring that data up and say, you know, you know, we’re, we’re seeing this type of bullying going on.

A student said the following:

Oh, yeah. Because it would let more people know that bullying has been going on and stuff.

#### Practicality and Adoption

All the school personnel (10/10, 100%) agreed that the program would be practical and workable, and almost all school personnel (9/10, 90%) stated that they would be likely to use the program at their school. For example, one school personnel member said the following:

...our students would definitely get on and be able to, you know, go through that program without any problems at all.

Another school personnel member stated the following:

Practical. I think that it addresses the needs of our students in their day-to-day interactions.

In terms of likelihood of use, one school personnel member stated the following:

I think it would be highly likely.

Another school personnel member added the following:

This will be helpful and they [schools] will use it because it wouldn’t take too much time away from the academics, academic goals that we have.

#### Teacher and Parent Training Modules

All school personnel (10/10, 100%) reported that a brief online teacher and parent training would be useful, and almost all (9/10, 90%) stated that it would provide a common language and a means for future collaboration. For example, one school personnel member shared the following:

It’s teaching the parents and the teachers what that looks like, you know, conversation or tools to help our kids.

Another school personnel member added the following:

Yes. Yes, so that you have this so that we’re all working together as a team. And using that common language.

#### Barriers

When asked about barriers to using the program, most school personnel members (7/10, 70%) reiterated that they would use the program, but many of them (8/10, 80%) discussed barriers. For example, one school personnel member stated the following:

I can’t think of one negative reason or one reason I wouldn’t want to use it.

However, other school personnel identified potential barriers, with one member stating the following:

Not being accessible on the devices that the kids have available.

Another school personnel member said the following:

Time. But I don’t see that as a factor for us because we could fit it into our advisory class.

A third school personnel member added the following:

Only thing I can think of is funding.

## Discussion

### Principal Findings

The purpose of this study was to examine the usability of the STAC-T application, a technology-based bystander bullying intervention designed specifically for middle schools in rural communities. We were interested in feedback from middle school personnel as they are in the position of making decisions related to adopting and implementing bullying interventions and from students as end users. The primary aim of this study was to test the usability of the STAC-T application and assess program utility, user-friendliness, and relevance as well as feasibility and ways to improve the program. Overall, both the quantitative survey results and qualitative interview findings indicate that participants perceived the STAC-T application to be useful, user-friendly, and appropriate for students at their schools and reported high levels of satisfaction with the program. The findings of this study indicate that the STAC-T application is relevant and feasible for implementation in middle schools in rural communities. The quantitative and qualitative findings are consistent with our previous usability testing research [40].

The findings of this study provide support for the usability of the STAC-T application. Both school personnel (mean 96.0, SD 3.9) and student (mean 88.6, SD 9.5) scores on the SUS demonstrated a very high level of usability, exceeding the standard cutoff score of 68 [51]. Both school personnel and students also rated the user-friendliness of the STAC-T application as very high, with all participants rating the program at ≥6 on a scale ranging from 0 to 7. We found no differences between school personnel and students on SUS scores or user-friendliness ratings, suggesting that both groups of users found the STAC-T application to be highly usable. The qualitative data supported these results, with both school personnel and students indicating that they perceived the program to be useful as well as relevant and appropriate for middle school students in rural communities. Furthermore, both school personnel and students reported high levels of satisfaction with the program, with 100% (10/10) of school personnel and 91% (10/11) of students indicating that they would recommend the program to others. Furthermore, most participants (20/21, 95%) gave the program 4 or 5 stars on a scale ranging from 1 to 5 stars. These findings are particularly important as usability and acceptability are associated with both program adoption and implementation [42].

In terms of practicality and adoption of the intervention, school personnel believed that their school would be likely to use the STAC-T application and identified cost, time, and access as potential barriers. Our results are aligned with those of previous studies that indicate that school administrators in rural communities feel favorably about adopting and implementing online programs to address the problem of bullying [20]. In addition, our findings are similar to those of previous studies that identify cost [7,20], time [56], and access to technology [57] as notable barriers to online programming implementation in schools located in rural areas.

School personnel and students reported positive perceptions of the STAC-T program, as well as feedback for program improvement. The results of the qualitative analyses showed that both school personnel and students thought that the STAC-T content was relevant and relatable and increased students’ knowledge of how to intervene in bullying situations. School personnel also stated that the program taught students empathy and prosocial attitudes. Furthermore, school personnel stated that the program was practical and workable and would be used at their school and that teacher and parent trainings would be useful additions to STAC-T to provide a common language among stakeholders. These findings suggest a need for bystander bullying intervention programs in rural schools that teach students how to intervene when they witness bullying behaviors through conducting role-plays to practice strategies across different bullying scenarios. In terms of program improvement, both school personnel and students highlighted the importance of having the entire program narrated to students. School personnel also talked about ways to improve user engagement by simplifying and slowing down a few program activities. Both students and school personnel also saw value in the posttraining tracker. Including these program modifications is likely to increase user engagement [40], which, in turn, can influence positive behavior change [47], potentially increasing the efficacy of STAC-T.

### Limitations

This study supports the usability, relevance, and feasibility of the STAC-T prototype. However, certain limitations must be noted. Participants were recruited from 2 schools in rural, low-income areas from 2 states, one in the Northwestern region and one in the Southern region of the United States. Although participants were recruited from 2 different states to increase generalizability, school personnel and students from different regions of the country may have a different perspective. Future research including a broader geographic sample would increase generalizability. Furthermore, most of the school personnel participants in this study were female, further limiting the generalizability of the study. In addition, because of the small sample size, we were unable to explore differences in demographic factors, limiting information that could guide improvements to the STAC-T application. Finally, it is also possible that social desirability influenced participants as they were aware that the goal of the study was to translate the in-person STAC intervention to a technology-based format.

### Implications

This study has important implications for the development and implementation of STAC-T in middle schools in rural communities. First, participants provided very high usability ratings for the STAC-T application, with qualitative data supporting the usability, utility, and relevance of the program. The findings also provided valuable information about the program itself, including the need for program narration and the utility of a type of bullying and strategy tracker that could be used after the training. School personnel also provided feedback about the importance of both teacher and parent modules to foster collaboration. These modules could be developed and offered to schools as companion modules for STAC-T. School personnel could provide teachers with the STAC-T teacher training during professional development time at school and provide parents with a link to the parent training via email. Training teachers, parents, and students could provide a common language and increase buy-in from all stakeholders [58,59]. In addition, participants indicated that program implementation is feasible as long as the program is cost-effective, brief, and students can access it on their school devices. Translating STAC from an in-person modality to an online platform can help decrease barriers to implementation, increasing implementation feasibility for rural schools by decreasing program costs and reducing the demand on schools by decreasing the need for staff training, in-class time, and expert support. Overall, the findings of this study provide valuable feedback and a strong scientific premise for moving forward with a large, multisite randomized controlled trial to examine the efficacy of the STAC-T application.

### Conclusions

Bullying is a significant public health concern for students in rural middle schools. Training students to intervene and developing programming that increases access are important factors in addressing this problem. The findings of this study demonstrate the usability, relevance, and feasibility of the STAC-T application. The preliminary data from this study support conducting a large, multisite randomized controlled trial to assess the efficacy of the STAC-T application for middle school students in rural communities. Technology-based bullying interventions, and STAC-T in particular, could be an instrumental approach to decreasing educational and mental health disparities in rural schools and helping address the problem of bullying.

## Abbreviations

SUS: System Usability Scale
